# A retrospective analysis of the therapeutic effects of 0.01% atropine on axial length growth in children in a real-life clinical setting

**DOI:** 10.1007/s00417-021-05254-5

**Published:** 2021-06-18

**Authors:** Hakan Kaymak, Birte Graff, Frank Schaeffel, Achim Langenbucher, Berthold Seitz, Hartmut Schwahn

**Affiliations:** 1 Internationale Innovative Ophthalmochirurgie GbR c/o Breyer Kaymak and Klabe Augenchirurgie, Duesseldorf, Germany; 2 Institute of Experimental Ophthalmology, Saarland University, Homburg, Germany; 3 Section of Neurobiology of the Eye, Ophthalmic Research Institute, University of Tuebingen, Tuebingen, Germany; 4 Department of Ophthalmology, Saarland University Medical Center UKS, Homburg, Germany

**Keywords:** Axial length, Children, Efficacy, Low-dose atropine, Myopia, Myopia control

## Abstract

**Background:**

Several randomized controlled studies have demonstrated the beneficial effects of 0.01% atropine eye drops on myopia progression in children. However, treatment effects may be different in a routine clinical setting. We performed a retrospective analysis of our clinical data from children to investigate the effect of 0.01% atropine eye drops on myopia progression in a routine clinical setting.

**Methods:**

Atropine-treated children were asked to instill one drop of 0.01% atropine in each eye every evening at 5 days a week. Myopic children who did not undergo atropine treatment served as controls. Objective refraction and ocular biometry of 80 atropine-treated and 103 untreated children at initial visit and 1 year later were retrospectively analyzed.

**Results:**

Myopic refractions in the treated and untreated children at initial visit ranged from −0.625 to −15.25 D (−4.21 ± 2.90 D) and from −0.125 to −9.375 D (−2.92 ± 1.77 D), respectively. Ages at initial visit ranged from 3.2 to 15.5 years (10.1 ± 2.7 years) in the treated and from 3.4 to 15.5 years (11.2 ± 3.0 years) in untreated children. Two-factor ANOVA for age and atropine effects on axial length growth confirmed that axial length growth rates declined with age (p<0.0001) and revealed a significant inhibitory effect of atropine on axial length growth (p<0.0015). The atropine effect on axial length growth averaged to 0.08 mm (28%) inhibition per year. Effects on refraction were not statistically significant.

**Conclusion:**

The observed atropine effects were not very distinctive: Statistical analysis confirmed that atropine reduced axial length growth, but to an extent of minor clinical relevance. It was also shown that beneficial effects of 0.01% atropine may not be obvious in each single case, which should be communicated with parents and resident ophthalmologists. 
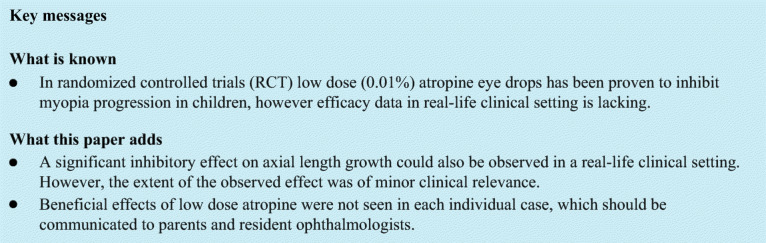

**Supplementary Information:**

The online version contains supplementary material available at 10.1007/s00417-021-05254-5.

## Introduction

Myopia is a common ophthalmic disorder that is primarily characterized by blurry distance vision if left uncorrected. With increasing severity of myopia, the risk increases for serious eye diseases which can cause irreversible blindness, like retinal detachment, choroidal neovascularization, and glaucoma [1–3]. It was estimated that 50% of the world population may be myopic by the year 2050, and that 10% may be highly myopic [4]. Also, it was observed that the prevalence of myopia is increasing [5] in Europe with high prevalence rates in younger people [6]. Therefore, there is an increasing need for treatment options to inhibit myopia progression.

In addition to optical therapies like orthokeratology, multifocal contact lenses (review [7]), or specially designed spectacle lenses [8], there is also the option of pharmacological interventions like with atropine eye drops. Topical atropine application can inhibit myopia progression at low doses with reduced side effects [9–12]. One goal of current randomized controlled trials (RCTs) is also to determine the optimal balance between side effects and myopia inhibition [13]. The official registry for clinical trials (clinicaltrials.gov) lists several RCT on low-dose atropine, some also in combination with other treatments for myopia. In a recent major study with 4 different doses of atropine, it was found that atropine is highly potent to reduce myopia progression in children, with a clear dose-dependent effect [14, 15]. It was also concluded that the optimal balance between side effects and myopia inhibition may be around 0.05%. Nevertheless, in the currently registered RCTs, the most commonly tested dose is still 0.01%.

Atropine represents a classical myopia intervention, already described in the mid of the 19th century by Cohn [16]. At that time, paralysis of accommodation was considered as a major mechanism by which atropine inhibits myopia, but this view has changed after it was found that atropine inhibits myopia also in avian models which have ciliary muscles not sensitive to atropine [17]. At present, the following mechanisms are discussed: (1) atropine stimulating dopamine release from the retina [18, 19] which represents an inhibitory signal for eye growth and myopia progression (review [20]), (2) atropine partially antagonizing adrenergic transmission by acting on alpha-2a receptors, in addition to muscarinic receptors [21], (3) atropine stimulating EGR-1 which is considered a growth-inhibiting signal for the eye [22, 23], (4) atropine causing choroidal thickening by relaxing non-vascular smooth muscles and/or stimulating choroidal blood flow [24], (5) a direct inhibitory effect of atropine on scleral growth [25, 26]. However, currently none of these mechanisms can be safely attributed to the inhibition of myopia [19, 27].

In the MVZ Makula-Netzhaut-Zentrum, Breyer Kaymak Klabe, in Duesseldorf, Germany, low-dose atropine eye drops (0.01%) are routinely offered to children with progressing myopia. Over the past 4 years, a large amount of data has accumulated on the potential therapeutic effects of this dose. Data were retrospectively analyzed to evaluate the effects of low-dose atropine treatment in a real-life clinical setting. To our knowledge, this is the first real-life study on the effects of 0.01% atropine on axial length growth in European children in everyday clinical routine.

## Methods

### Patient pool

Atropine treatment was offered as the individual decision of the treating ophthalmologist. Atropine treatment was recommended, if at least one of the following criteria had been fulfilled: (1) cycloplegic refraction <+0.75 D at the age of 7, (2) myopia progression >0.5 D in the last year, or (3) axial length growth >0.22 mm. There was no upper limit of myopia or myopia progression to receive atropine treatment. Nevertheless, many myopic patients (i.e., their parents) decided not to undergo atropine treatment.

**Fig. 1:**
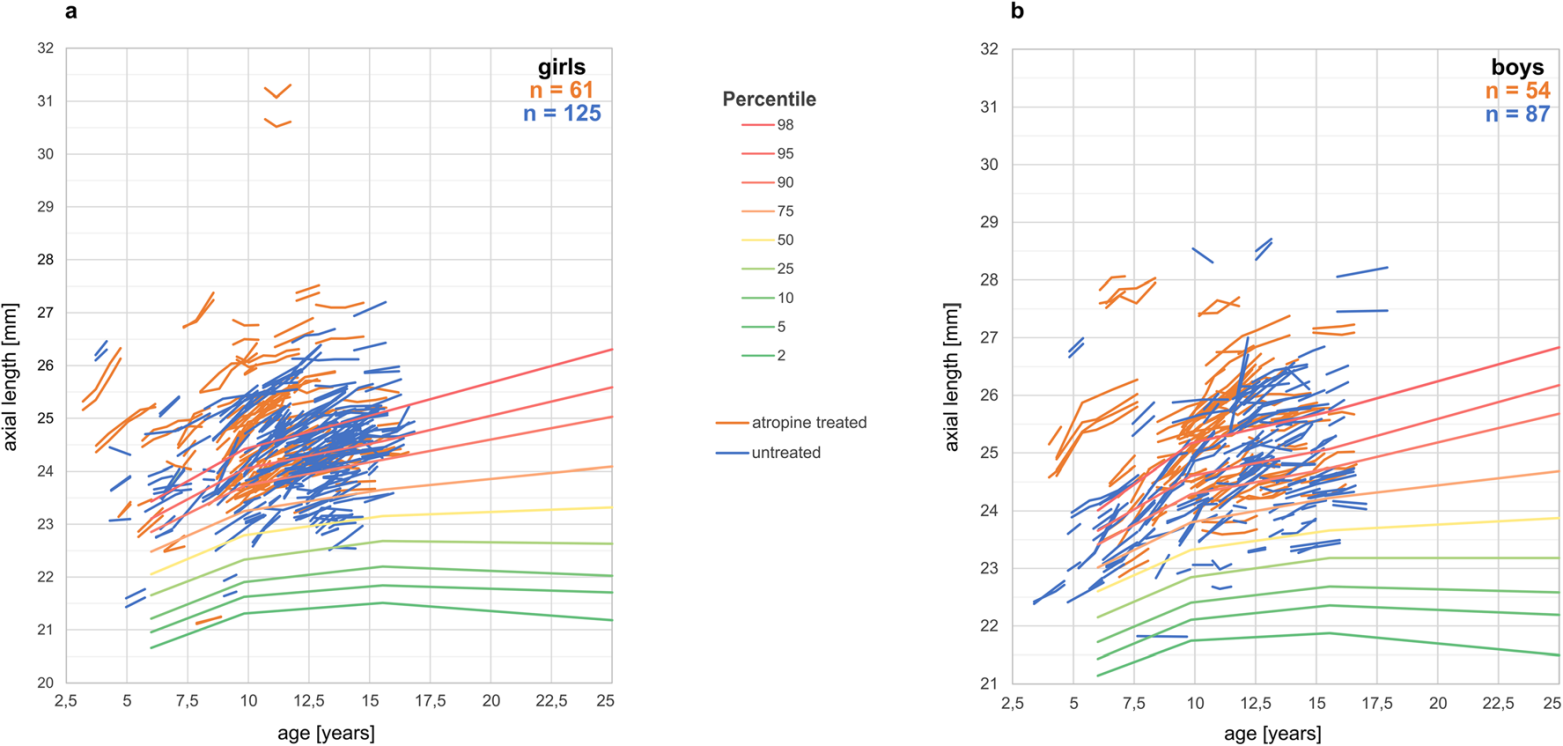
Development of axial length in girls (**a**) and boys (**b**), plotted against age. Orange lines denote atropine-treated myopic children, blue lines myopic children who did not receive atropine treatment. The continuous colored lines represent the percentiles for developing myopia as determined by Tideman et al. [28]

In the past 4 years, 163 children started low-dose atropine (0.01%) treatment in our clinic. Among those, 115 children have completed at least one follow-up visit including measurement of refraction and axial length after having started the atropine treatment (Fig. 1). Since late 2017, axial length measurements have been routinely included in the examination also in children who do not undergo atropine treatment. Therefore, axial length data were available for 212 untreated children, measured at multiple visits, so that their axial length growth could serve as reference.

Because the intervals between follow-up visits in both the atropine-treated and the untreated group were highly variable, data were analyzed only from those children where the interval between baseline and a follow-up visit was between 9 and 15 months. Those intervals are referred to below as “12-month interval,” even though these were not exactly 12 months. However, the calculated myopia progression rates were determined based on the exact time difference between the two visits.

Neither of the groups had received any other mode of myopia control before being monitored in this study or received any additional treatment to inhibit myopia progression during the course of the 12-month observation period.

### Procedures and examinations

Upon the baseline visits, a detailed anamnesis took place including (among others) general diseases; medication; family history; comprehensive eye examination including wide-angle fundus picture, OCT imaging, and fundoscopy; and an orthoptic examination. Subsequently, cycloplegic or non-cycloplegic objective refraction was performed (KR-800S, Topcon, Tokyo, Japan), as well as subjective refraction with trial lenses. Cycloplegia was only carried out in children suspected of showing accommodation during refraction. In addition, ocular biometry was performed (IOL-Master 700, Carl Zeiss Meditec, Jena, Germany). Also, static and dynamic pupil sizes were measured; however, for the present study, only data on ocular biometry (axial length, lens thickness, anterior chamber depth, and corneal curvature) and refraction (spherical equivalents) are analyzed.

### Treatment instructions

Patients were asked to apply a single atropine eye drop in each eye every evening, for 5 days a week. The diluted atropine solution was mainly obtained from one supplier (98% from BergApotheke, Tecklenburg, Germany). Control visits including refraction and axial length measurement were suggested every 6 months. New spectacle corrections were recommended when myopia had progressed by more than ≥ 0.5 D and a noticeable improvement of distance visual acuity was achieved with a new correction. Parents were advised to supervise the viewing habits of their children, like viewing distances during reading, reading time, and availability of sufficient illuminances. Parents were also advised to encourage children to play outside as often as possible.

### Adverse side effects

Patients’ records were searched for potential adverse side effects.

### Data analysis

Changes in spherical equivalent and axial length were normalized to an exact 12-month interval (mm/year or D/year, respectively) by taking refraction or axial length differences between the first and the later visit and dividing it by the intermittent time interval, as measured in months, and then multiplied by 12. In the plots, the abscissa shows ages in the middle between the two visits. For the statistical analysis, a two-factor ANOVA was used on to separate age and treatment effects.

### Ethics

This retrospective analysis was carried out in accordance with the Declaration of Helsinki. Before analyzing the collected data in anonymized form for scientific purposes, informed (parental) consent was obtained both for atropine-treated children and untreated children as required for ethical approval. The analyzed data was routinely collected during treatment; there were no additional interventions or examinations performed.

## Results

### Patient sample characteristics

**Fig. 2:**
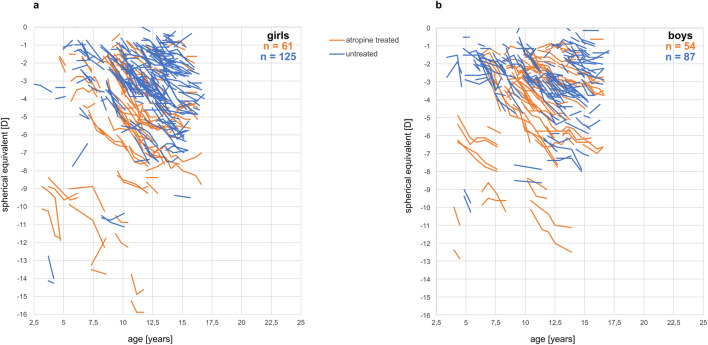
Development of refractions in girls (**a**) and boys (**b**), plotted against age. Orange lines denote atropine-treated myopic children, blue lines myopic children who did not receive atropine treatment

Figures 1 and 2 show an overview on the development of axial lengths and refraction in all myopic children monitored in the clinic (115 atropine-treated and 212 untreated). Axial length data at baseline (initial visit) and follow-up visits are plotted into the reference graph provided by Tideman et al. [28] which is based on normative data from more than 12,000 European children (Fig. 1). According to their reference plot, most of the children were in the high-risk group to develop high myopia, based on their axial lengths. Inspection of axial length growth rates in atropine-treated children (orange lines) and untreated children (blue lines) does not reveal obvious differences. It can be seen that treatment durations and the number of visits were highly variable. Therefore, our data analysis was limited to those children where data for a follow-up visit at 12-month interval were available (12.7 ± 1.4 months in the atropine-treated group and 12.5 ± 2.1 months in the untreated group). After applying this exclusion criterion, data from only 80 atropine-treated and 103 untreated children remained in the sample. Their average ages, initial refractions, and axial lengths are shown in Table 1. Note that on average, children in the atropine group were younger, more myopic, and had longer eyes. One girl with an axial length above 30 mm (see Fig. 1) was considered an outlier and was excluded.

**Table 1:** Characteristics of children who had two visits with an interval of about 12 months. This exclusion criterion reduced the numbers of children to n=80 in the atropine-treated group and to n = 103 in the untreated group

	Atropine-treated	Untreated
Age (year)		
Average (SD) Median (range)	10.1 (2.7) 9.9 (3.2 to 15.5)	11.2 (3.0) 11.9 (3.4 to 15.5)
Spherical equivalent [D]		
Average (SD) Median (range)	−4.21 (2.9) −3.19 (−0.625 to −15.25)	−2.92 (1.77) −2.75 (−0.125 to −9.375)
Axial length [mm]		
Average (SD) Median (range)	24.82 (1.33) 24.58 (21.11 to 31.24)	24.40 (0.97) 24.46 (21.43 to 26.94)

*SD* standard deviation

### Evaluation of the treatment effects of atropine

**Fig. 3:**
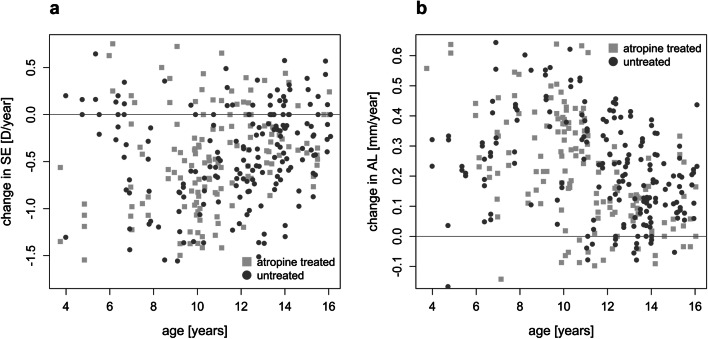
Average changes in spherical equivalents (SE) (**a**) and axial length (AL) (**b**), referenced to the first 12-month interval. Light gray squares refer to atropine-treated, dark gray circles to untreated children. Each symbol denotes one eye

**Fig. 4:**
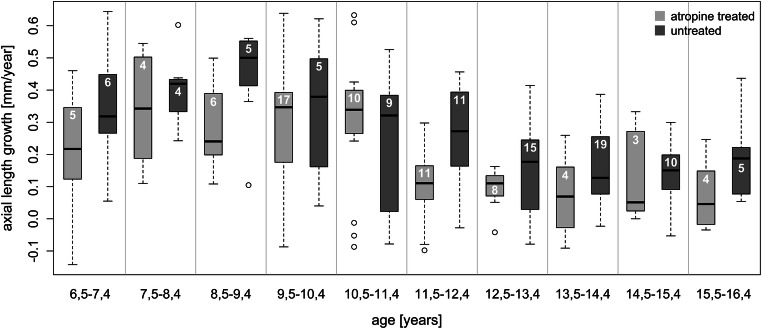
Axial length growth rates per year in atropine-treated (light gray columns) and untreated children (dark gray columns), analyzed in age bins of 12 months. White numbers indicate the numbers of contributing children. Note that eyes grew generally faster at younger ages, but the effects of atropine were not obviously age-dependent (confirmed by two-factor ANOVA, see text)

To evaluate the potential beneficial effects of low-dose atropine on myopia progression in this sample (Table 1), the annual increase in axial length (Fig. 3a) and the annual progression of myopia (Fig. 3b) were plotted for each subject as described in the “Methods” section. Statistical analyses were demanding due to the fact that children in the untreated group were, on average, slightly older and less myopic, with shorter axial lengths, than in the atropine-treated group (Table 1). Figure 4 shows the changes in axial length growth within the 12-month interval in atropine-treated and untreated children in 1-year age bins. It can be seen that axial length growth was faster in most bins for the untreated children. Two-factor ANOVA revealed that axial growth rate declined with age (p<0.0001), and inhibition of axial length growth by atropine treatment was significant (p<0.0015), independently of age (the factor age*treatment effect was not significant). Averaging the differences in axial length growth between atropine-treated and untreated children for each age group reveals an inhibition of 0.08 mm per year in the atropine-treated group, equivalent to an average of 28% reduction in axial length growth. Changes in axial length growth were also summarized in only three age ranges in order to compare to other studies (see Online Supplementary Information Figure 1). The effects of atropine on refraction (spherical equivalent) were not significant (data not shown).

### Myopia progression rates

Regarding the changes in spherical equivalent, 58% of atropine-treated eyes progressed mildly (by less than −0.5 D/year), 30% progressed moderately (by −0.5 to −1.0 D/year), and 12% progressed severely (by more than −1 D/year). For untreated eyes, the respective proportions were 57%, 31%, and 12%. In the case of axial length, 51% of atropine-treated eyes displayed a mild progression of less than 0.2 mm/year, 26% progressed moderately by 0.2 to 0.35 mm/year, and 23% progressed heavily by more than 0.35 mm/year. For untreated eyes, the respective proportions were 47%, 28%, and 25%.

### Effects of atropine treatment on anterior chamber depth, lens thickness, and corneal curvature

**Fig. 5:**
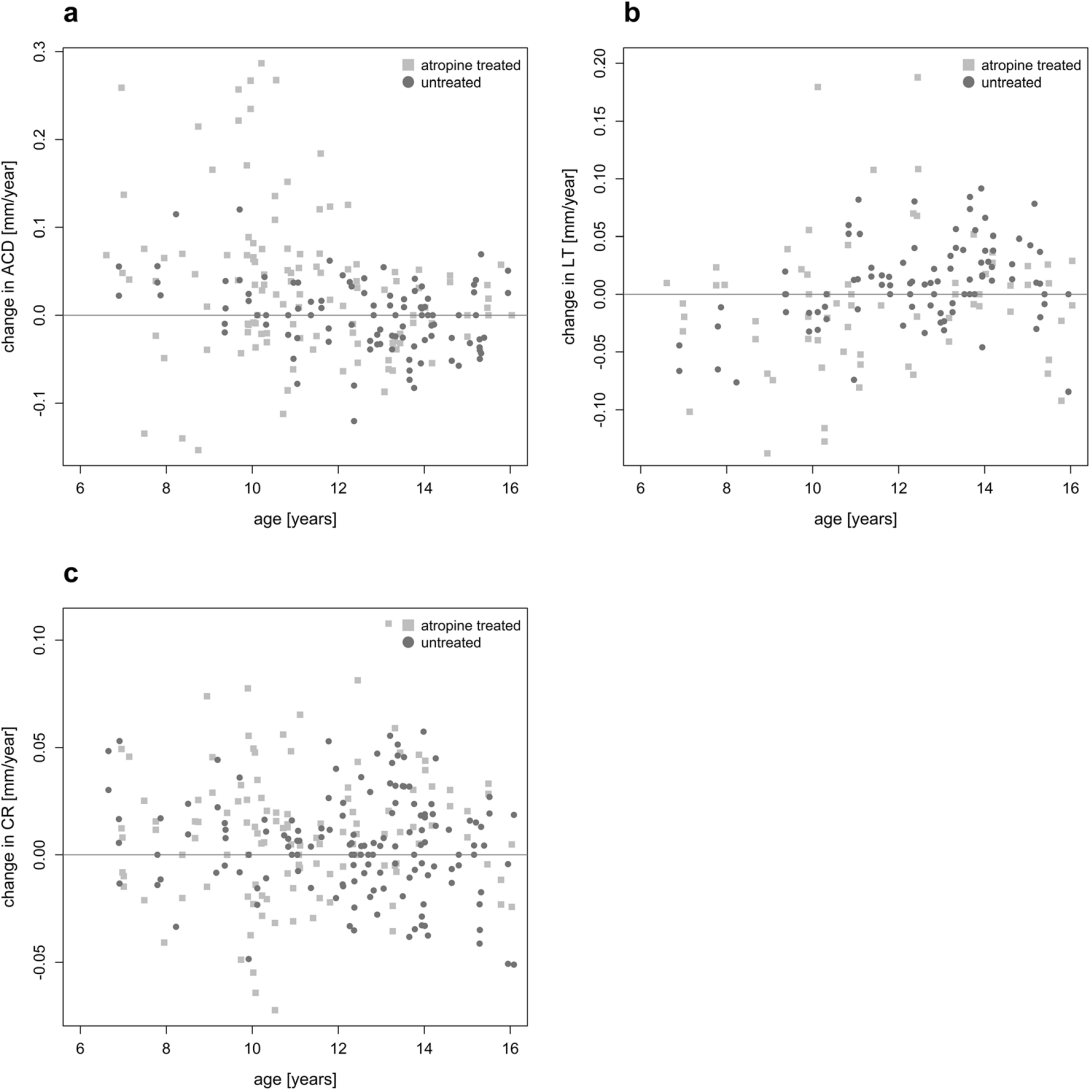
Average changes in anterior chamber depth (ACD) (**a**), lens thickness (LT) (**b**), and corneal radius of curvature (CR) (**c**), referenced to the first 12-month interval. Light gray squares refer to atropine treatment, dark gray circles to untreated children. Each symbol denotes one eye

To find out whether atropine may interfere with the normal development of the anterior segment in children, we also analyzed anterior chamber depth (Fig. 5a), lens thickness (Fig. 5b), and corneal curvatures (Fig. 5c). It can be seen that all three variables changed by less than 0.1 mm, with no clear age dependence. There were no significant differences between atropine-treated and untreated control eyes.

### Evaluation of self-reported adverse side effects

In total, 16.7% of the atropine-treated children reported adverse side effects of atropine eye drops. Those were (sorted by incidence) burning eyes after applying the eye drops (9.7%), pupil dilation persisting into the following instillation day (5.6%), photophobia (5.6%), and redness (5.6%). One child reported to have difficulties to read at short distances (15 cm), and one child reported the need for more frequent and stronger blinking. Some children reported combinations of those effects, but none reported serious complications.

## Discussion

Our retrospective analysis of myopia progression data from children treated with low-dose atropine eye drops is in contrast to randomized controlled trials (RCT). In our study, data were collected from everyday clinical routine, children were not randomized, treatments not age-matched, and also not matched by baseline refractions. Since this supposedly reflects the situation in most European clinics, we believe that this is an important aspect to observe more closely. What can be expected from low-dose atropine treatment in an everyday clinical routine? It is already known for other ophthalmic treatments that observational trials under real-life conditions reveal inferior outcomes compared to controlled treatment trials [29]. As we see here, this also applies to current atropine treatments.

### Evaluation of the treatment effects of atropine

In our group of myopic children, we found that the effects of low-dose (0.01%) atropine eye drops were not very conspicuous in a real-life clinical setting. Beneficial effects of low-dose atropine were not seen in each individual case. This was also reported by Yam et al. [15]. Significant effects of atropine were only detected when the entire sample of myopic children with and without atropine treatment were compared. We found a significant inhibitory effect on axial length growth, which was, contrary to what Li et al. [30] found, not dependent on age. A correlation between baseline axial length and changes in axial length after 1 year of atropine treatment could not be observed (see Online Supplementary Information Figure 2).

Different from large RCTs where 0.01% atropine eye drops caused a highly significant inhibition of myopic progression (i.e., ATOM2 study, LAMP study), our study showed an inhibitory effect only on axial lengths and not on spherical equivalents. This might be partially attributed to the fact that refractions were not consistently performed under cycloplegia. Furthermore, in those children, who actually received cycloplegia, the cycloplegic effect might have varied. However, axial length data are more representative to determine the treatment effects of atropine since atropine also fully relaxes the ciliary muscle and therefore may cause more hyperopic refractions. Brennan et al. [31] proposed that the “cumulative absolute reduction of axial elongation” represents an optimal metric to quantify effect sizes of treatments for myopia. There is also agreement that axial length is the more relevant predictor for pathological changes in the fundus that are associated with high myopia.

Atropine effect becomes also visible in myopia progression rates, since atropine-treated children, that where on average younger and more myopic, showed same myopia progression in terms of change in spherical equivalent and change in axial length as untreated children, that were older and less myopic.

### Optimal “low dose atropine concentration” for myopia control?

In the present analysis, eye drops containing atropine sulfate at a concentration of 0.01% were used for the myopic children, based on results of the ATOM2 study. This is currently the most widely used atropine concentration for myopia intervention as can be seen from the registry of clinical trials (clinicaltrials.gov). Also, a recent RCT in Beijing in 76 atropine-treated and 83 placebo-treated children aged 6–12 years found a reduction in axial length growth by only 22%, using 0.01% atropine eye drops (p = 0.004) [32]. The significance level was similar to what we found in our analysis (p = 0.0015). However, Khanal and Philips [33] have recently concluded after re-analyzing data of the ATOM2 study [12] and the LAMP study [15] that there is only little evidence that this dose had a significant effect on axial growth. They even argue that treatment with 0.01% atropine might delay the implementation of an effective dose in a myopic child and that 0.025% or 0.05% should be used instead. The authors of the LAMP study [15] pointed out that there was a clear dose-dependency of the effect of atropine on myopia progression over 2 years, with two times higher efficacy of 0.05% atropine compared to 0.01%. Other studies used much higher doses of atropine, for example, 0.5% atropine [34]. Recently, Klaver and Polling [35] confirmed that 0.5% atropine eye drops had been successfully used in The Netherlands now for one decade. They found a reduction in axial length growth with atropine by up to 0.2 mm/year in 74% of the children over 3 years. Twelve percent of the children had axial length growth rates of 0.2 to 0.3 mm/year and 14% more than 0.3 mm/year. Despite the dose-dependency of atropine effects on myopia progression, also adverse side effects depend on the dose. Accordingly, the higher the atropine dose, the more adverse side effects like photophobia and reading problems occur. Such side effects may prompt more patients to drop out of the treatment.

### Inconsistency of atropine effect in different studies

There are various possible reasons for the variable effects of low-dose atropine in different studies:

1. Compliance may vary among studies. In Rotterdam, [35] 73% adherence to the treatment regimen was reported, which is impressive given the high doses of atropine and the related side effects. In the study in Beijing [32], parents were asked to mark the dates of medication in a calendar. The protocols suggested more than 80% compliance rate, but it may be difficult to prove that the reports were reliable. In the current study, we cannot exclude the risk of sub-optimal compliance. While it was explained to the parents that regular atropine application was necessary to generate a reliable effect on myopia progression, it was not controlled other than asking the parents on the occasion of the visit. In fact, some random checks as to how much atropine was left over in their supplies and how often new prescriptions were picked up led to the suspicion that atropine application was not as regular as pretended.
2. A potentially uncontrolled variable is the exact formulation of the atropine dilutions across different studies. Typically, dilutions are obtained from pharmacies, but users often do not exactly know their composition and pH values or whether atropine maintains its potency under the applied conditions. The pH values are potentially relevant already since 1957; Kondritzer and Zvirblis [36] argued that “Atropine is most stable at a pH between 3 and 6 and, in our experience, compounding pharmacies pay little attention to this when diluting an existing solution.” On this topic, see also Saito et al. [37]. It would be interesting to find out whether higher doses could be replaced by lower dose atropine solutions with optimized formulations.
3. The current data are retrospective and cross-sectional which means that neither refractions nor ages were properly matched. Additionally, neither range of baseline refraction nor range of baseline axial length of both groups is typical for what can be found in RCTs. Both groups contained children that showed a higher myopia progression rates as compared to the literature (see Figs. 1 and 2). However, we believe that it is also relevant to illustrate the real-life effects of atropine in an everyday clinical routine because this represents the reality of low-dose atropine application for myopia control in children. At least, the current study achieved a similar significance of atropine effects on axial length as a recent RCT with similar subject numbers [32].

### Variability of the data

Inspecting the raw data on myopic progression and axial length changes (Fig. 3), the question arises as to how variable the measurements may be. There are a number of children in which myopia regressed over 1 year by up to 0.75 D and axial length became even shorter. While the regression in refractions could be explained by different levels of cycloplegia, axial length data depend on the accuracy of the measurement device. Data were collected with the IOL-Master which provides a warning message if six measurements show too much variability. Such measurements were discarded and repeated. We, therefore, consider it unlikely that measurement noise is responsible for the apparent reductions in axial length. Another possible reason is variable fixation during the measurements, which may have caused higher levels of variability in repeated measurements, without generating a warning message to discard the measurement.

### Adverse side effects

The used atropine eye drops with a concentration of 0.01% seemed to be very well tolerated, and the rate of children that reported any adverse side effect was low. There were also no systemic adverse side effects. During atropine treatment, pupil dilatation may be most relevant since it may generate photophobia. However, not all children that observed pupil dilation also experienced photophobia. We observed a low rate of photophobia of 5.6%, comparable to Wei et al. [32], and even slightly lower than what Sacchi et al. [38] reported in their retrospective analysis. There was no need to prescribe photochromatic or multifocal glasses. Only one child reported inacceptable side effects which resulted in termination of the treatment.

Our study showed that a significant but small effect of low-dose atropine on axial length growth was detected also in a non-controlled and randomized controlled study. However, its effects were not very obvious under these conditions, and this should be transmitted to the parents to avoid exaggerated expectations. Our study also suggests that there may be factors that need to be better controlled which are (1) uncertain compliance and (2) varying composition of atropine eye drops with potential variations in potency. Observations of long-term effects of low-dose atropine treatment alone or in combination with other myopia-inhibiting strategies in real-life settings are underway.

## Supplementary information

ESM 1 Axial length growth rates per year in atropine treated (light gray columns) and untreated children (dark gray columns), analyzed in age bins of 3 and 4 years. White numbers indicate the numbers of contributing children (PNG 4645 kb)

High Resolution (TIF 74233 kb)

ESM 2 Change in axial length of atropine-treated children depending on axial length at baseline for different age bins (PNG 6193 kb)

High Resolution (TIF 99009 kb)
