# Supplementary figures and images for: A retrospective analysis of the therapeutic effects of 0.01% atropine on axial length growth in children in a real-life clinical setting
Source: Graefes Arch Clin Exp Ophthalmol. 2021 Jun 18;259(10):3083–92. doi: 10.1007/s00417-021-05254-5 (PMC8478763; doi:10.1007/s00417-021-05254-5)

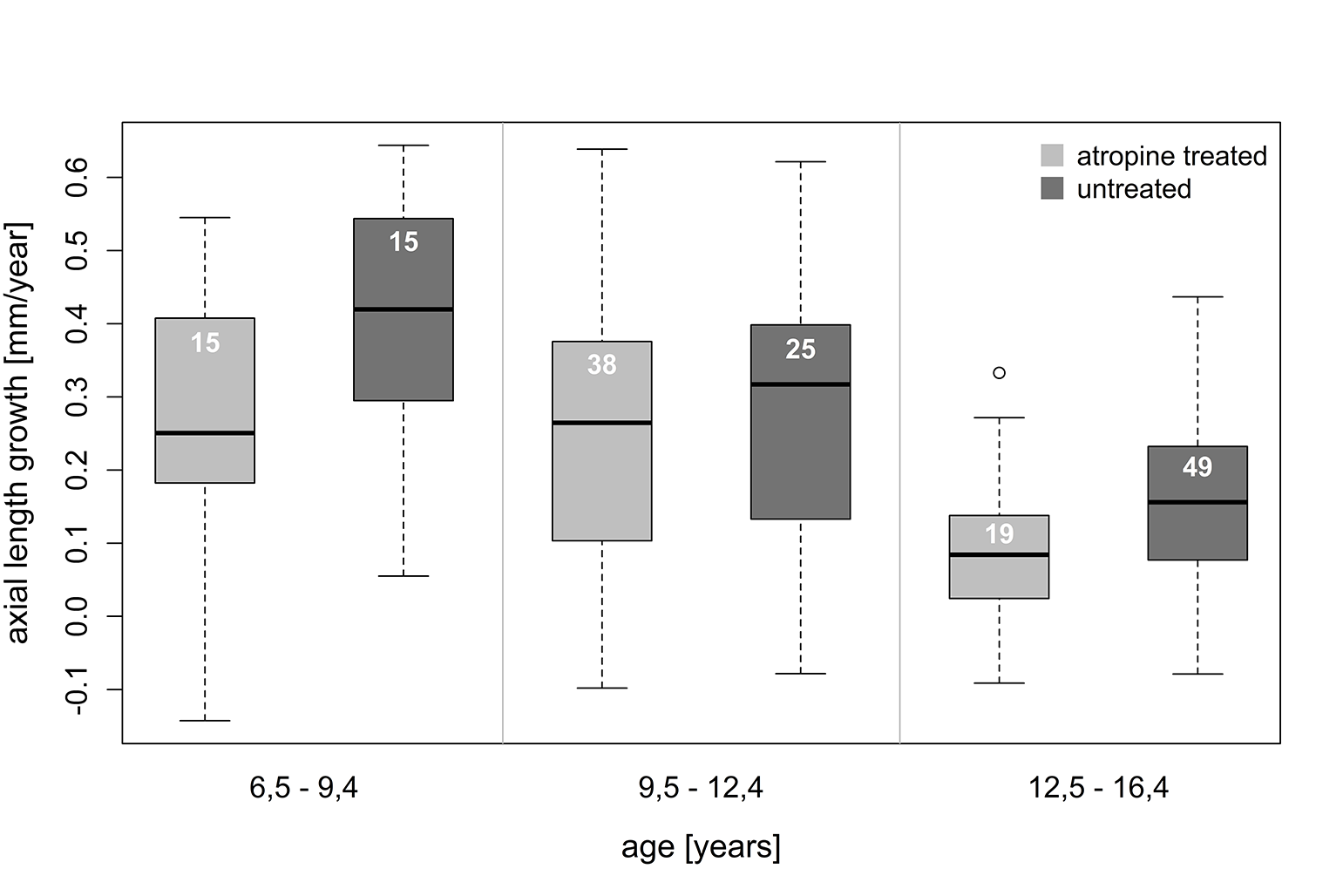

Supplement: Supplementary file 1 — Axial length growth rates per year in atropine treated (light gray columns) and untreated children (dark gray columns), analyzed in age bins of 3 and 4 years. White numbers indicate the numbers of contributing children (PNG 4645 kb) [file 417_2021_5254_Fig1_ESM.png]

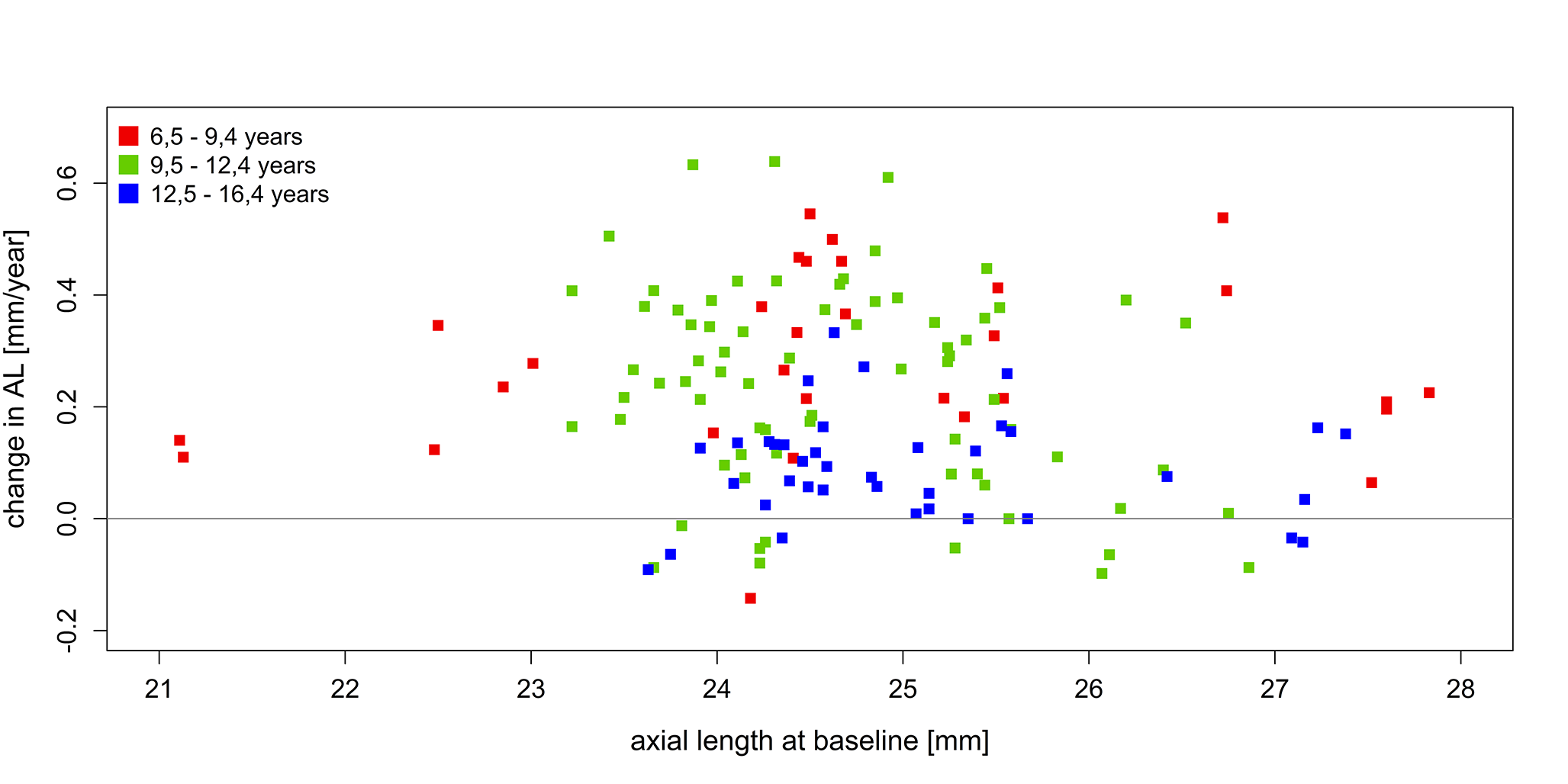

Supplement: Supplementary file 3 — Change in axial length of atropine-treated children depending on axial length at baseline for different age bins (PNG 6193 kb) [file 417_2021_5254_Fig2_ESM.png]
